# A Delayed Diagnosis of Myxedema Coma

**DOI:** 10.7759/cureus.33370

**Published:** 2023-01-04

**Authors:** Carla Williams

**Affiliations:** 1 Geriatrics, Harvard Medical School, Boston, USA

**Keywords:** uncontrolled, severe, hypothyroidism, decompensated, myxedema coma

## Abstract

A 58-year-old man without any personal or familial cardiac history presented to the emergency department with complaints of worsening left-sided chest pain that began at rest, described as a burning sensation and escalating to a 9/10 severity. He denied any personal or familial cardiac history but admitted that he had not been medically evaluated in approximately five years. His physical examination was notable for obesity, xerosis, macroglossia, and bilateral lower extremity edema. His initial labs demonstrated critical troponin levels that peaked at 11.5 ng/mL and he was diagnosed with a myocardial infarction and underwent cardiac catheterization with percutaneous stenting of the left anterior descending artery. His post-operative period was complicated by prolonged lethargy that was determined to be myxedema coma two days later when his thyroid stimulating hormone level was found to be 78 mIU/mL.

## Introduction

First introduced in London, England, in 1879, myxedema coma is the term still used to describe severe, decompensated, or uncontrolled hypothyroidism. The term originated during a time when hypothyroidism was referred to as myxedema, a Greek word used to describe the “doughy” appearance of the skin [1]. The condition is triggered by a stressful event and can be potentially fatal [2]. Demographically, those affected are typically elderly women. Epidemiologically, the precipitating event is usually an infection, myocardial infarction, or an acute cerebrovascular event. Still, other etiologies can include hypoglycemia, hypothermia, congestive heart failure, gastrointestinal bleeding, trauma and even some medications such as anesthetics, beta-blockers, diuretics, lithium, rifampin and amiodarone [2,3].

The hallmarks of myxedema coma include altered mental status, hypothermia, hypotension, hypoventilation, and bradycardia, among a plethora of other signs and symptoms. Moreover, presentations can differ extensively, with every patient exhibiting a unique amalgam of the aforementioned abnormalities with varying severities. Despite the term, however, not every patient with myxedema coma is comatose on presentation leading to the delayed recognition of this life-threatening event [4]. There is a need for improved terminology to better capture the varied manifestations of uncontrolled or untreated severe hypothyroidism, such as decompensated hypothyroidism [5].

Finally, even when successfully identified, special considerations must be made regarding treatment when the inciting event is cardiac in nature or the patient has a significant cardiac history, as thyroid hormones affect cardiac contractility and systemic vascular resistance. Particularly after myocardial infarction, IV levothyroxine can potentially cause the rapid restoration of metabolic homeostasis, increasing the strain on cardiac function and the risk of arrhythmias and further myocardial damage [6].

## Case presentation

The patient was a 58-year-old male brought to the emergency department via ambulance at roughly 7 AM to evaluate worsening left-sided chest pain. The patient reported that the pain began at 2 AM while working on his computer. He described the pain as a burning sensation or tightness in his left chest that radiated to his back between his shoulder blades and was associated with numbness in his arms. After the initial onset of the pain, the patient attempted to go to bed; however, the pain escalated to a 9/10 severity. He alerted his wife, who called an ambulance. Before emergency medical services (EMS) arrived, the patient chewed a 325 mg aspirin tablet. When EMS arrived, he received nitroglycerin, and his chest pain severity was reduced to 7/10. 

Upon arrival to the emergency department, along with chest pain, the patient reported chronic bilateral lower extremity swelling over the past year and chronic dyspnea with exertion for at least six months prior. Because of these symptoms, the patient, who is generally sedentary, ambulates with a cane. The patient denied any cardiac history but reported being a former smoker who quit five years prior. He had not been medically evaluated in at least five years and denied any prior surgical history. His family history was notable for Type II diabetes mellitus. Socially, the patient lived with his wife and daughter. He worked from home as a painter and was trying to establish an online business that required him to spend most of his time on the computer. He did not engage in any exercise. He had a 61.5-pack-year smoking history before quitting five years before and endorsed a history of alcohol use disorder but stated that he quit 20 years prior. He also admitted to experimenting with cocaine in the past but denied any drug use at present.

A review of systems was notable for fatigue, weakness, daytime somnolence, loss of appetite, weight gain, chronic dyspnea, bilateral lower extremity edema, constipation, abdominal distension, cold intolerance, hair loss, chronic back pain, and bilateral upper extremity numbness. 

**Figure 1:**
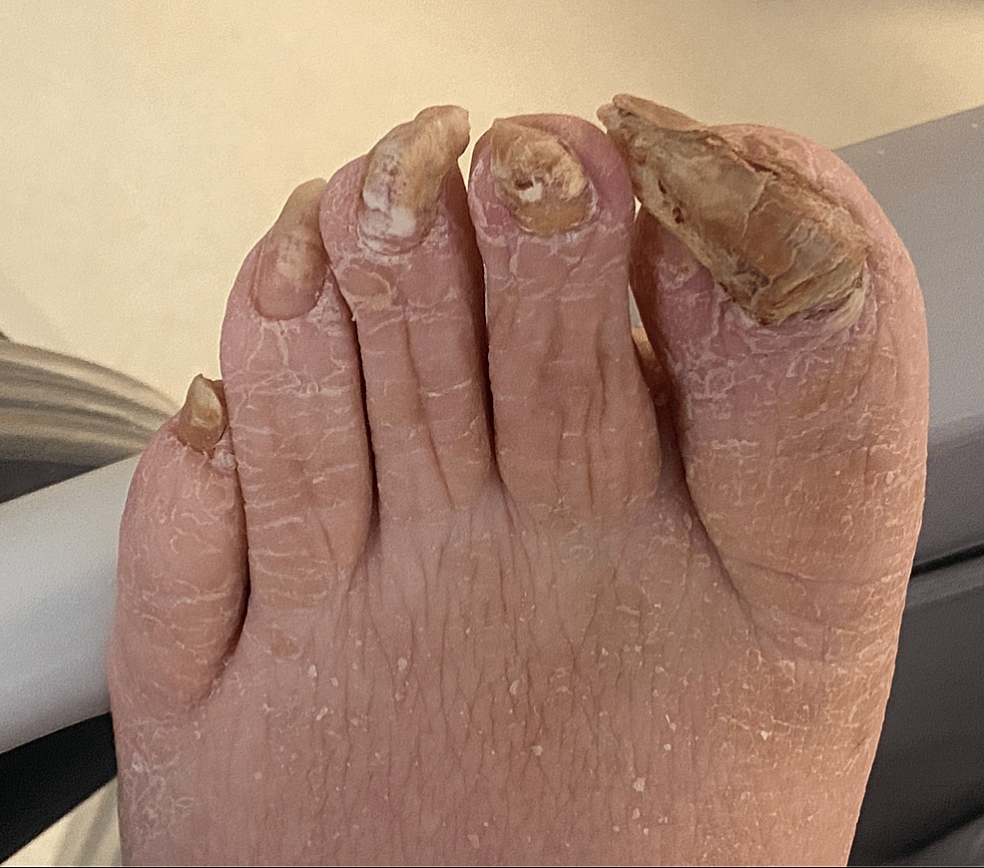
Image of the patient's overgrown toenails and xerosis.

**Figure 2:**
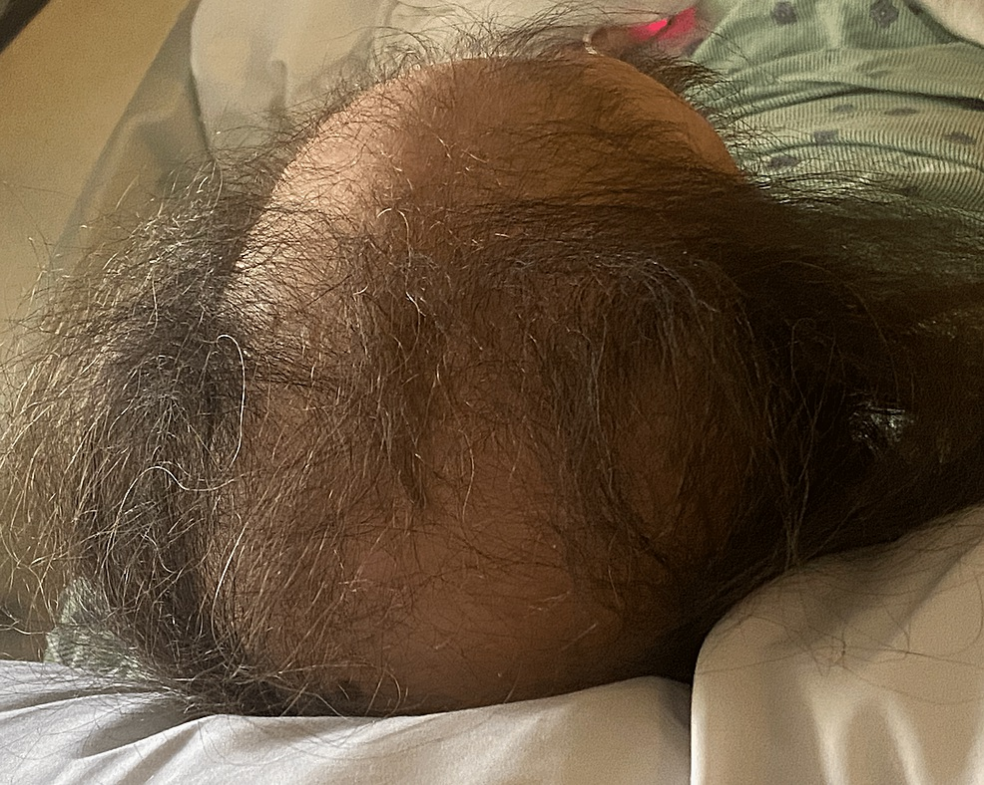
Image depicting the patient's hair loss.

Vitals on admission were temperature 37^ o^C, blood pressure 102/70, heart rate 62, respiratory rate 14, and oxygen saturation (SpO_2_) 95% on 2L nasal cannula (NC). Physical examination revealed an obese middle-aged man with a flat affect who appeared older than his stated age. He had poor dentition with the wiring of the hard palate, macroglossia, xerosis, cool doughy skin, thick overgrown nails (Figure 1), and thin, coarse hair (Figure 2). There were rales on auscultation of lung fields. Heart sounds were distant, and he had diminished peripheral pulses and cool extremities. 2+ pitting lower extremity edema bilaterally was noted, along with hypoactive bowel sounds. 

Chest x-ray demonstrated bilateral infiltrates and cardiomegaly consistent with heart failure. Lab values were pertinent for critical troponin of 8.14 ng/mL that peaked at 11.5 ng/mL. The patient was taken for cardiac catheterization within 48 hours which demonstrated ejection fraction of 20%, 90% stenosis of the proximal left anterior descending artery (LAD), and 80% stenosis of the mid LAD. Percutaneous stenting to the LAD was performed during the procedure. Post-procedure, the patient was noted to be more somnolent with decreased SpO_2_ (in the high 80s) despite 6L NC. Arterial blood gas (ABG) on non-rebreather was 7.278/71.7/72/32, and after 6L NC was 7.351/58/68/32, consistent with respiratory alkalosis, likely from retained carbon dioxide. The critical care team was contacted for evaluation of respiratory status.

On evaluation by the critical care team, the patient was noted to be easily arousable to voice but was otherwise sedated. His pupils were equal but sluggishly reactive. He had decreased breath sounds bilaterally with dullness to percussion over the bilateral bases and poor inspiratory effort. On cardiac examination, he had a normal heart rate and regular rhythm, significant nonpitting edema in the lower extremities and upper forearms bilaterally. Compression wraps were in place on his lower extremities. No focal neurological deficits were observed. Pertinent laboratory tests prior to transfer to the intensive care unit are listed in Table 1 below. 

**Table 1:** Pertinent laboratory values prior to transfer to the intensive care unit (ICU).

Significant laboratory values	Prior to transfer to the intensive care unit	Reference range
Thyroid-stimulating hormone	78 mIU/L	0.5 - 5 mIU/L
T3-Uptake	26%	24% - 37%
Thyroxine	1.2 mcg/dL	4.5 - 11.2 mcg/dL
Free thyroxine index	0.31 mcg/dL	4.8 - 12.7 mcg/dL
Free thyroxine	0.15 ng/dL	0.7 - 1.9 ng/dL
Cortisol	24 mcg/dL	5 - 25 mcg/dL

Endocrinology was consulted, and the patient was treated for myxedema coma. The treatment of myxedema coma involves supportive care with fluids, vasopressors, ventilation if necessary, passive warming, and glucocorticoids for potential concurrent adrenal insufficiency, followed by thyroid hormone replacement. Thyroid hormone replacement starts IV with 200-400 mcg daily for three to five days, followed by a daily oral dose based upon typical dosing of 1.6 mcg/kg (the IV dose is 75% of the oral dose) [3,5-8]. Steroids are usually given as 100 mg IV hydrocortisone every eight hours until serum cortisol results are obtained and may be discontinued if random cortisol is greater than 18 mg/dL [3,5-8].

This patient was loaded with levothyroxine 50 mcg IV for three days, then transitioned to maintenance 100 mcg by mouth once per day due to his recent myocardial infarction. With a normal random cortisol level, there was no indication for pretreatment with hydrocortisone as the patient was not deemed at risk of adrenal insufficiency. In the meantime, he continued on goal-directed medical therapy for his coronary artery disease.

Repeat echocardiogram status post percutaneous stenting was significant for ejection fraction of 25 - 30%, akinesis of the apex, severe global hypokinesis of the apical and anterior segments, and persistent moderate pericardial effusion. The patient was therefore fitted with a LifeVest. He participated in inpatient rehabilitation but could not afford a skilled nursing facility, so he was eventually discharged home with home health services.

## Discussion

One of the core factors in myxedema coma is longstanding hypothyroidism, although not necessarily previously diagnosed. Due to our patient's low contact and follow-up with medical providers, he likely had compensated hypothyroidism until his inciting event. Typical classical symptoms of hypothyroidism that usually lead to medical consultation are fatigue, constipation, weight gain, cold intolerance, voice changes, coarse hair, and dry, pale, and cool skin; however, with age, patients' symptoms can be atypical such as changes in mobility such as in this case with the patient relying on a cane [3,5,7,8].

Key physical exam findings in myxedema coma are altered mental status, alopecia, bladder dystonia, blood pressure changes (diastolic hypertension early followed by hypotension and bradycardia), dry, cool skin, hypothermia, and hypoventilation, with altered mentation being the biggest suggestion to consider diagnosis rather than a comatose state [3,5,7,8]. Lab abnormalities include anemia, elevated creatine phosphokinase, elevated creatinine and transaminases, hyperlipidemia, hypoglycemia, hyponatremia, and hypercapnia and hypoxia on arterial blood gas measurements [3,5,7,8]. In such cases, the diagnosis of myxedema coma can be made with an elevated thyroid stimulating hormone (TSH) level and low free thyroxine [3,5,7,8].

Unfortunately, the differentials for such signs and symptoms are broad. Furthermore, due to similar abnormalities and the precipitating event, myxedema coma may be underdiagnosed or identified late, such as in this case. Ultimately, the term myxedema coma does not encapsulate the plethora of presentations of decompensated hypothyroidism and can delay its recognition. There is a need for improved terminology more reflective of the spectrum of manifestations of severe hypothyroidism to improve the identification of this disease and patient outcomes [5].

Finally, with regard to the treatment of decompensated hypothyroidism in patients with acute myocardial infarction, a balance must be struck. Too high of a dose can precipitate a fatal arrhythmia and worsen the damage; however, too low of a dose will be insufficient to reverse the severe effects of decompensated hypothyroidism [9]. Furthermore, treatment with thyroxine (T4) may be less effective due to impaired conversion of T4 into triiodothyronine (T3), which commonly occurs during severe illness and inadequate caloric intake [9]. Hypothyroidism generally has harmful effects on the cardiovascular system, including increased systemic vascular resistance, while cardiac output, heart rate, and myocardial contractility are decreased [6].

While several studies address the management of decompensated hypothyroidism, unfortunately, there is limited clinical evidence to help guide the treatment of decompensated hypothyroidism in acute myocardial infarction [6]. One retrospective study demonstrated that most patients with severe hypothyroidism (quantified as a TSH of 10 mIU/L or higher) are more likely to be treated with only oral levothyroxine, which was associated with higher 30-day mortality and new/worsening heart failure. This is because the restoration of euthyroidism has been shown to have cardioprotective effects such as positive cardiac remodeling and even reduction in the myocardial infarction area [6]. This study concluded that the ideal management is the slow up-titration of IV levothyroxine in such circumstances [6]. 

## Conclusions

Key findings in myxedema coma include alopecia, bladder dystonia, blood pressure changes, dry, cool skin, hypothermia, and hypoventilation, with altered mentation being the biggest suggestion to consider diagnosis rather than a comatose state. There is a need for improved terminology more reflective of the spectrum of manifestations of severe hypothyroidism to improve the identification of this disease and patient outcomes.

Moreover, while there are guidelines in place to manage decompensated hypothyroidism, with limited clinical studies performed in the presence of acute myocardial infarction, more research is required to help guide therapy in cases such as this one. However, this case serves as an example of the benefit of IV levothyroxine dosing even in such circumstances and also brings attention to the positive effects of addressing severe hypothyroidism early in the event of acute myocardial infarction such as improved cardiac remodeling and shrinkage of the infarction area. 
